# Comparative Transcriptomics Analysis Reveals Genes Associated with a Dehiscent-Corolla Mutant in Sesame (*Sesamum indicum* L.)

**DOI:** 10.3390/ijms262411841

**Published:** 2025-12-08

**Authors:** Xiaoxu Feng, Weifei Yang, Hengchun Cao, Qin Ma, Ming Ju, Weixiu Hou, Cong Mu, Pengjie Chang, Yinghui Duan, Zhanyou Zhang, Guiting Li, Qiuzhen Tian, Haiyang Zhang, Hongmei Miao

**Affiliations:** 1 Henan Sesame Research Center, Henan Academy of Agricultural Sciences, Zhengzhou 450002, China; bbxe2013@163.com (X.F.); yangweifei4115@163.com (W.Y.); chczhima@163.com (H.C.); maqin073130@163.com (Q.M.); jumingzz@163.com (M.J.); houweixiu92@163.com (W.H.); mucong0313@163.com (C.M.); changpengjie2025@163.com (P.C.); duanyly@aliyun.com (Y.D.); zhangzhanyou2025@163.com (Z.Z.); liguiting07111106@163.com (G.L.); tianqiuzhen@163.com (Q.T.); 2 Key Laboratory of Specific Oilseed Crops Genomics of Henan Province, Henan Sesame Research Center, Henan Academy of Agricultural Sciences, Zhengzhou 450002, China; 3 College of Agronomy, Henan Agricultural University, Zhengzhou 450002, China

**Keywords:** transcriptomics, floral organ development, differentially expressed genes, WGCNA, sesame

## Abstract

Floral organ development plays a critical role in determining crop yield and quality, yet the molecular mechanisms underlying this process in sesame remain poorly understood. In this study, comparative transcriptomic analyses were conducted between the wild-type Yuzhi11 and a dehiscent-corolla mutant, css1, to investigate the genetic basis of floral organ variation. A total of 114 differentially expressed genes (DEGs) were identified between the two genotypes in both the main and lateral flowers, among which 47 candidate genes were implicated in main/lateral floral organ development. A weighted gene co-expression network analysis (WGCNA) revealed two gene modules significantly associated with the mutant’s main and lateral flowers, respectively. Five hub genes were identified within each module, and their potential regulatory networks and protein–protein interactions networks were characterized. These findings provide new insights into the genetic regulation of flower and fruit development in sesame, and may inform future breeding strategies to optimize floral and yield related traits.

## 1. Introduction

Sesame (*Sesamum indicum* L., 2n = 26), a member of the genus *Sesamum* in the family *Pedaliaceae*, originated from tropical Africa and is one of the world’s oldest oilseed crops, with a cultivation history of over 5000 years [1]. Sesame seeds contain 50–55% oil, of which 80–85% comprises unsaturated fatty acids, and 20–30% protein. It is also rich in calcium, polyphenols, sterols, vitamin E, and essential amino acids. Its unique compounds, like sesamin and sesamolin, exhibit anti-cancer, hepatoprotective, and antioxidant properties, supporting sesame’s broad applications in food and pharmaceutical industries [2]. The normal inflorescence structure and floral organ development are crucial determinants of crop yield and quality. Therefore, exploring the molecular mechanisms underlying sesame flower development holds both theoretical and practical significance for improving yield stability and productivity.

In flowering plants, floral organ differentiation typically follows the pattern of “sepals–petals–stamens–carpels” pattern. The classical ABCDE model explains the genetic regulation of this process: class A and E genes regulate sepal development; class A, B, and E genes govern petal formation; class B, C, and E genes specify stamen and ovule development; and class C and E genes control carpel development [3,4,5]. Among numerous transcription factors involved in flower development, MADS-box genes play a dominant role [6]. The ABCDE model genes contain various MIKC-type MADS transcription factors-encoding genes, including *Apetala1* (*AP1/FUL*, class A), *Apetala3* (*AP3/PI*, class B), *Agamous* (*AG*, class C), *Seedstick-like* (*STK*, class D), and *Sepallata* (*SEP*, class E) [4,7,8,9]. Beyond these canonical regulators, additional MADS-box genes also contribute to floral morphogenesis. For example, the B-class gene *GLOBOSA* (*GLO*) is involved in petal and stamen development [10]. In *Physalis floridana*, loss of *GLO* function transforms the corolla and stamens transforming into sepals and carpels, respectively. The DOLL1-encoded protein, a GLO homolog, binds distinct CArG-box motifs in the *PFCRC* promoter, exerting opposite effects on carpel identity and floral organ size [11]. In rice, the B-class gene *OsMADS16/SPW1* acts epistatically to the C2H2-type zinc finger gene *SRO*, and together with the C-class genes *OsMADS3* and *OsMADS58*, regulates pistil and stamen development and floral meristem determinacy [12]. Another MADS box protein, AGL6, suppresses nectary and petal formation in the outer and ventral floral regions. Meanwhile, TCP transcription factors CYC1/2/3 define dorsal and ventrolateral identities and, along with AP3, AGL6, and the MYB transcription factor DIV1, regulate floral symmetry and patterning [13,14,15,16].

Despite these insights from model plants, studies on sesame flower development remain still limited. Completion of the sesame genome has enabled functional genomic research progress to a new stage [17]. Genome-wide structural variation and association analyses have identified *SiCEN2,* a homolog of *TFL1*, as a key regulator maintaining vegetative growth and inflorescence meristem identity. *SiCEN2* plays a central role in apical determinate inflorescence formation, single-stem architecture, and the typical sesame flower pattern of one main and two lateral flowers per leaf axil [18,19,20,21]. Additionally, the photoperiod-related gene *SiCOL1* (a homolog of *AtCO*) regulates flowering time, enhancing the geographic adaptability of sesame cultivars [22]. The bHLH gene *SibHLHA* (a homolog of *AtTT8*) regulates anthocyanin synthesis and, by interacting with WER-like or TTG1 proteins, contributes to the purple corolla phenotype in sesame cultivar Ganzhi9 [23]. In our previous study, through a GWAS (genome-wide association study) analysis, Li et al. discovered that the alternative splicing of the *SiGIF1* gene led to abnormal development of sesame petals and capsules, as well as smaller seeds in wild-type sesame cultivar Yuzhi11 and the dehiscent-corolla mutant css1 [24]. GIF1 ((growth-regulating factor) GRF-interacting factor) is a transcriptional co-activator of the plant-specific growth-regulating factor (GRF). This gene exhibits pleiotropic effects and is involved in regulating the development of plant organs, such as stems, leaves, and flowers, among others, as well as the process of seed formation. For example, induced by exogenous cytokinin, the upstream flowering-related gene *PbRR1* (two-component response regulator) can activate the transcription of *PbGIF1*, affecting cell proliferation and the determination of floral meristems, thereby altering plant height or internode length, organ size, and organ morphology [25]. In addition, AtSOD7 inhibits the interaction between AtGIF1 and AtGRF by competitively interacting with AtGIF1, thereby restricting the growth of organs and seeds [26]. Li et al. also searched homolog GIFs in sesame, *Arabidopsis*, and rice, respectively, and found GIF1 is functionally conserved in plants [24,27]. However, the molecular mechanisms and gene regulation network controlling the morphological development of sesame floral organs remain largely unexplored.

In this study, we analyzed the dehiscent-corolla mutant css1, generated by ethyl methanesulfonate (EMS)) mutagenesis, and its wild-type counterpart Yuzhi11 [24]. To elucidate the molecular mechanisms underlying corolla development in sesame, we conducted comparative transcriptomic analyses of both the main and lateral flowers from both genotypes, along with RNA-Seq profiling of different css1 tissues. The primary objective was to identify the key differentially expressed genes (DEGs) potentially involved in corolla development, and to construct co-expression networks significantly associated with floral organ formation. Through a weighted gene co-expression network analysis (WGCNA), we aimed to uncover critical gene modules and hub genes, thereby providing theoretical insights into the molecular regulatory networks governing floral organ development in sesame.

## 2. Results

### 2.1. Transcriptomic Data Assessment

**Figure 1:**
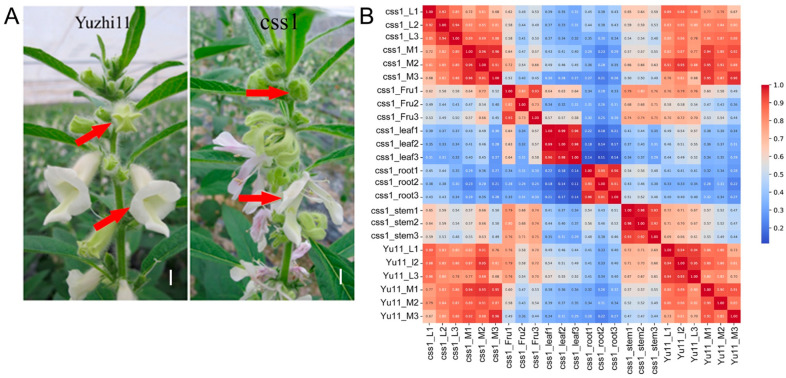
Phenotypic differences between Yuzhi11 and css1 flowers and transcriptomic data correlation. (**A**) Floral phenotypes of Yuzhi11 (wild type) and the css1 mutant. The red arrows indicate closed and open flowers. Scale bar = 1 cm. (**B**) Correlation heatmap of transcriptomic data from various samples. “L” and “M” denote the lateral and the main flower, respectively, and “Fru” represents the fruit. The numbers 1, 2, and 3 represent three biological replicates.

Sesame flowers develop in the leaf axils, typically consisting of one central main flower flanked by two lateral flowers. In the wild-type variety Yuzhi11, all three flowers are bell-shaped and normally develop into capsules at maturity. By contrast, the dehiscent-corolla mutant css1 exhibits split, strip-shaped petals in all flowers (Figure 1A). Only the main flower of css1 produces malformed capsules with smaller seeds, while the lateral flowers fail to form capsules.

To investigate gene expression changes associated with petal development, we performed a comparative transcriptomic analysis of the main and lateral flowers from Yuzhi11 (wild type) and css1. Additionally, RNA-seq libraries from the root, stem, leaf, and capsules tissues of css1 were constructed and sequenced to explore the tissue-specific expression patterns. Three biological replicates were included for each material.

After removing adapters and low-quality reads, a total of 1,077,356,452 cleaning reads (161.6 Gb) were obtained, averaging 44,889,852.17 reads and 6.73 Gb data per sample (Table S1). The clean reads showed GC content ranging from 45.62% to 48.75% and Q30 between 97.47% and 97.93%, indicating high sequencing quality. Alignment rates to the sesame reference genome ranged from 73.14% to 77.33% across the samples. Correlation heatmaps confirmed high reproducibility and strong consistency among biological replicates (Figure 1B).

Together, these results demonstrated that the RNA-seq data were of high quality and suitable for identifying DEGs and constructing gene co-expression networks.

### 2.2. Corolla-Specific Expression Gene Analysis

To identify genes specifically expressed in corollas, transcriptomic data from multiple tissues of the css1 mutant were analyzed. Genes were defined as corolla-specific when showing no expression in non-floral tissues (FPKM = 0) and detectable expression (FPKM > 1) in either the main or lateral flowers. Based on this criterion, 66 genes were specifically expressed in the lateral flowers and 98 in the main flowers. Among them, 55 genes exhibited expression (FPKM > 1) in both the main and lateral flowers, suggesting their potential roles in corolla development in the css1 mutant (Table S2). The remaining genes, expressed exclusively in either the main or lateral flowers, may participate in early floral meristem differentiation and lateral organ development.

### 2.3. DEG Analysis Between Yuzhi11 and the Dehiscent-Corolla Mutant css1

To explore the molecular mechanisms underlying floral and petal development, we compared gene expression profiles between the main and lateral flowers of Yuzhi11 and css1, respectively. Four comparison groups were established: css1 vs. Yuzhi11 main flower (cs_yz_M), css1 vs. Yuzhi11 lateral flower (cs_yz_L), css1 main vs. lateral flower (cs_M_L), and Yuzhi11 main vs. lateral flower (yz_M_L). A total of 621, 1482, 1921, and 312 DEGs were identified in cs_yz_M, cs_yz_L, cs_M_L, and yz_M_L groups, respectively. Among them, 266 and 729 genes were upregulated, while 355 and 753 genes were downregulated in the cs_yz_M and cs_yz_L comparisons groups, respectively (Tables S3 and S4).

**Figure 2:**
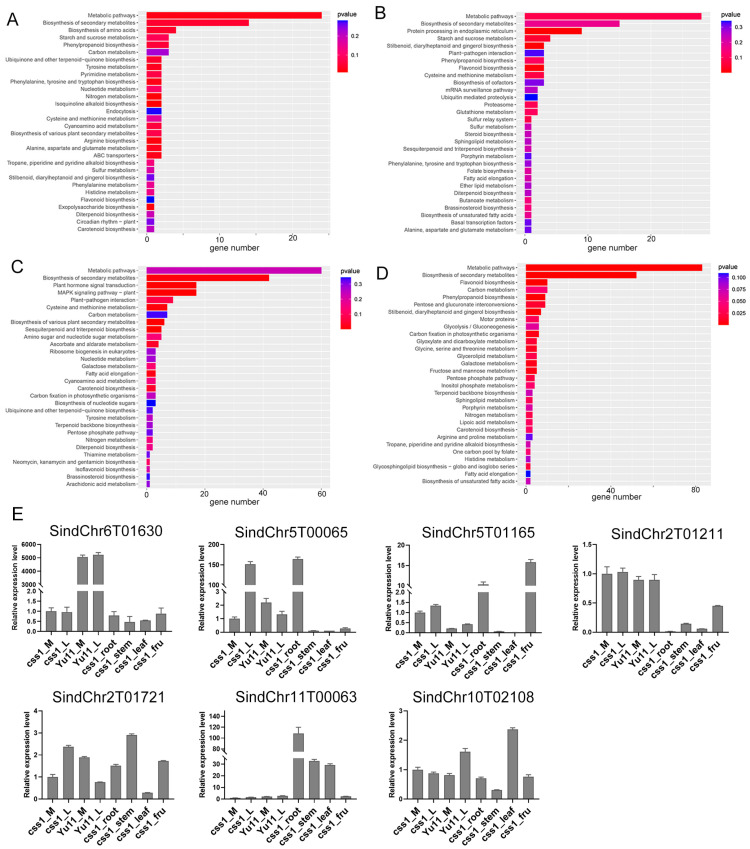
KEGG enrichment and qRT-PCR validation of DEGs in cs_yz_M and cs_yz_L comparison groups. (**A**,**B**) KEGG pathway enrichment analysis of upregulated (**A**) and downregulated (**B**) DEGs in the cs_yz_M group. (**C**,**D**) KEGG pathway enrichment analysis of upregulated (**C**) and downregulated (**D**) DEGs in the cs_yz_L group. (**E**) qRT-PCR validation of selected DEGs across different tissues and genotypes. The endogenous *SiTub1* gene was used to normalize the expression levels of DEGs, data are provided as mean ± SDs.

A gene ontology (GO) enrichment analysis revealed that upregulated DEGs in the cs_yz_M group were mainly enriched in “protein import into nucleus” and “amino acid metabolic process”, whereas downregulated DEGs were associated with “proton transmembrane transport” and “methylation”. In the cs_yz_L group, the upregulated DEGs were enriched in biological processes of “defense response” and “DNA-templated transcription”, whereas downregulated DEGs were primarily involved in “carbohydrate metabolic process” and “pectin catabolic process” (Figure S1). A Kyoto Encyclopedia of Genes and Genomes (KEGG) pathway enrichment analysis showed that both the upregulated and downregulated DEGs in the cs_yz_M and cs_yz_L groups were predominantly enriched in “metabolic pathways” and “biosynthesis of secondary metabolites pathways” (Figure 2A–D), suggesting that an altered secondary metabolism may contribute to the petal mutation phenotype in css1.

To validate the RNA-seq data, several DEGs were randomly selected for qPCR verification. The qPCR results were consistent with the transcriptomic data, confirming the reliability of the DEG analysis (Figure 2E, Table S2).

### 2.4. Analysis of DEGs Associated with the Dehiscent-Corolla Phenotype

To identify the genes potentially responsible for the dehiscent-corolla phenotype, DEGs from the cs_yz_M and cs_yz_L comparison groups were further analyzed. A total of 114 overlapping DEGs were identified, including 75 downregulated and 39 upregulated genes in the css1 mutant (Table S5). Among these, 37 genes lacked functional annotation, suggesting they may represent sesame-specific or uncharacterized genes. Among the downregulated DEGs, two genes encoded a cyclic nucleotide-gated ion channel (CNGC4) and a RanBP1 domain-containing protein, respectively. In addition, several genes related to the auxin signaling pathway were differentially expressed between css1 and Yuzhi11, including those encoding auxin efflux carriers and auxin-responsive proteins (Tables S3 and S4), indicating that auxin signaling may be disrupted in css1 and contribute to the dehiscent-corolla phenotype.

A comparison between the main and lateral flowers revealed striking expression differences. The yz_M_L group showed only 312 DEGs, whereas the cs_M_L group exhibited 1921 DEGs (Tables S6 and S7). Only 47 DEGs were shared between the two groups (Figure S2), suggesting distinct developmental programs between the main and lateral flowers in the css1.

**Figure 3:**
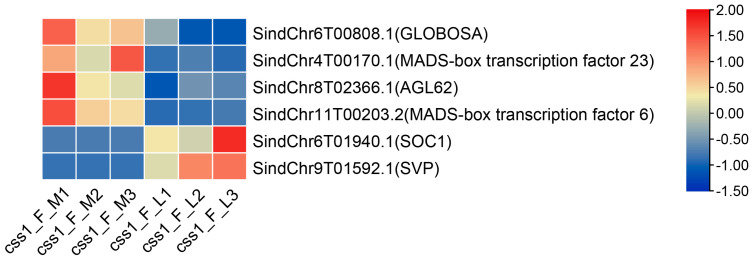
Expression heatmap of *MADS-box* genes among DEGs in the cs_M_L group. “L” represents the lateral flower and “M” represents the main flower. The numbers 1, 2, and 3 represent three biological replicates.

Several DEGs in the cs_M_L group belonged to the MADS-box gene family (Figure 3), known as regulators of floral organ development [6]. For example, *GLOBOSA* was significantly upregulated in the css1 main flowers (Figure 3). In *Antirrhinum*, *GLOBOSA* mutations resulted in sepaloid petals and carpelloid stamens [10]. Another MADS-box gene, *AGL62*, associated with endosperm development [28], showed a higher expression level in the css1 main flowers than in the lateral flowers (Figure 3). Similarly, *MADS6* and *MADS23* were upregulated in the css1 main flowers (Figure 3). Their rice homologs, *OsMADS6* and *OsMADS23,* regulate floral organ identity and tillering, respectively [29,30]. By contrast, two flowering-related genes, *Suppressor of Overexpression of Constans 1* (*SOC1*) and *Short Vegetative Phase* (*SVP*), were upregulated in the lateral flowers [31]. These altered gene expressions potentially contributed to the failure of capsule formation in these flowers.

### 2.5. Gene Co-Expression Network Analysis

**Figure 4:**
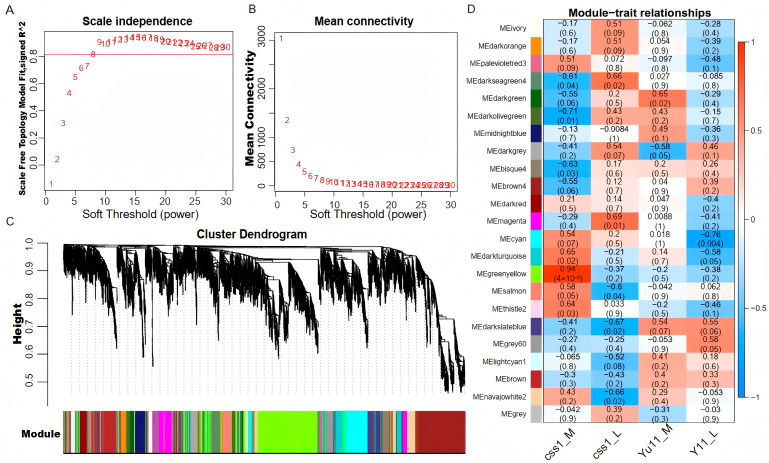
Weighted gene co-expression network analysis (WGCNA). (**A**,**B**) Determination of the soft-thresholding power: scale-free topology fit index (**A**) and mean connectivity (**B**) for various powers. (**C**) Gene clustering dendrogram based on topological overlap. Each leaf represents a single gene. A total of 22 distinct co-expression modules were identified and labeled with different colors. (**D**) Heatmap showing the correlation between modules and sample traits. In each cell, the top number indicates the Pearson correlation coefficient, and the bottom number represents the corresponding *p*-value.

**Figure 5:**
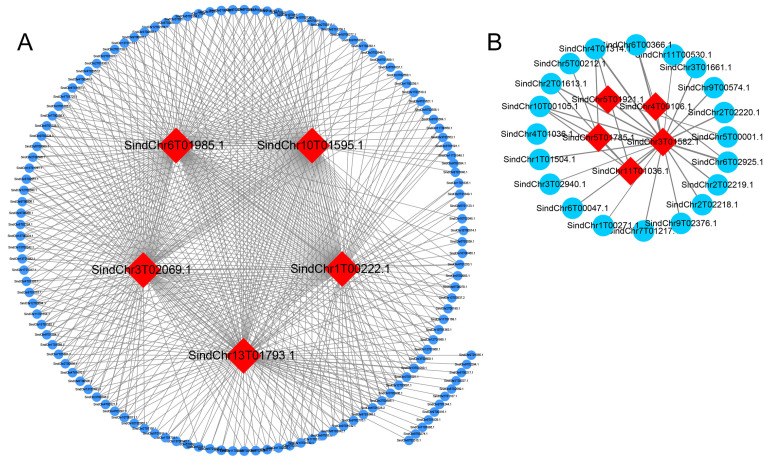
Co-expression network analysis. (**A**) Co-expression network of the five hub genes in the MEgreenyellow module. (**B**) Co-expression network of the five hub genes in the MEmagenta module. Hub genes are indicated by red diamonds. Dark blue and light blue circles represent the co-expression network genes in MEgreenyellow and MEmagenta module, respectively.

To further identify the genes potentially associated with the dehiscent-corolla phenotype, a WGCNA was performed using transcriptomic data from the main and lateral flowers of both the Yuzhi11 and the css1 mutant. Genes with FPKM ≥ 1 in at least one sample and a coefficient of variation > 0.5 at expression levels were retained, resulting in 15,274 genes for analysis. A soft-threshold power of eight was selected to achieve scale-free topology and optimal mean connectivity (Figure 4A,B). A total of 22 co-expression modules were identified and labeled with different colors (Figure 4C). A correlation analysis between the modules and the sample phenotypes revealed two modules significantly associated with the css1 mutant (Figure 4D). The MEgreenyellow module was strongly correlated with the css1 main flowers (r = 0.94, *p* = 4 × 10^−6^), and the MEmagenta module was significantly correlated with the css1 lateral flowers (r = 0.69, *p* = 0.01). These two modules contained 708 and 98 genes, respectively. The five genes with the highest intramodular connectivity were identified as hub genes within each module (Table 1). In the MEgreenyellow module, hub genes encoded a BURP domain-containing protein 3-like, pathogenesis-related protein STH-2-like, mitochondrial uncoupling protein 5, major allergen Pru ar 1-like, and thaumatin-like protein. In the MEmagenta module, three hub genes encoded metallothionein-like protein, NADH dehydrogenase [ubiquinone] 1 beta subcomplex subunit 10-B-like, and ESSS subunit of NADH:ubiquinone oxidoreductase (Complex I) protein, while two lacked annotation. The top 100 co-expressed genes for each hub gene were visualized in Cytoscape based on weighted correlation values (Figure 5, Tables S8 and S9). A GO analysis of the genes in the MEgreenyellow co-expression network showed significant enrichment in biological processes, such as “regulation of DNA-templated transcription”, “protein phosphorylation”, and “transmembrane transport”. The most represented molecular function terms were “protein binding”, “DNA binding”, and “ATP binding”, whereas cellular component terms were fewer and primarily related to “membrane”. By contrast, only three genes in the MEmagenta module had GO annotations, enriched in molecular functions, including “proton transmembrane transporter activity”, “iron–sulfur cluster binding”, “nucleic acid binding”, and “zinc ion binding”. These gene were primarily associated with “proton transmembrane transport” in the biological process, and “proton-transporting two-sector ATPase complex” in the cellular component (Tables S10 and S11).

### 2.6. Expression Analysis and Interaction Network Prediction of Hub Genes

**Figure 6:**
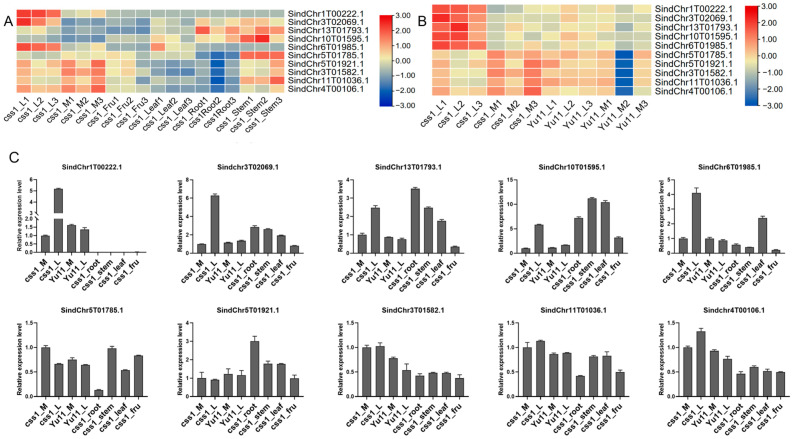
Expression analysis of hub genes. (**A**) Heatmap showing the expression patterns of 10 hub genes in various tissues of the css1 mutant. (**B**) Heatmap of hub gene expression in the main and lateral flowers of the css1 mutant and Yuzhi11. “L” represents the lateral flower, “M” represents the main flower, and “Fru” represents the fruit. The numbers 1, 2, and 3 represent three biological replicates. (**C**) qPCR-based expression profiles of selected hub genes across different tissues and in both css1 and Yuzhi11. The endogenous *SiTub1* gene was used to normalize the expression levels of hub genes, data are provided as mean ± SDs.

Among the ten identified hub genes, *SindChr13T01793.1*, *SindChr10T01595.1*, and *SindChr5T01785.1* were highly expressed in stems, while the remaining seven genes showed preferential expression in the main or lateral flowers of the css1 mutant (Figure 6A). Notably, all ten hub genes exhibited higher expression levels in both the main and lateral flowers of css1 compared with those of Yuzhi11 (WT) (Figure 6B).

A qPCR analysis confirmed these expression patterns, showing consistency with the RNA-seq data (Figure 6C). These results suggest that the identified hub genes are likely involved in the formation of the dehiscent-corolla phenotype and the development of lateral floral organs in css1.

**Figure 7:**
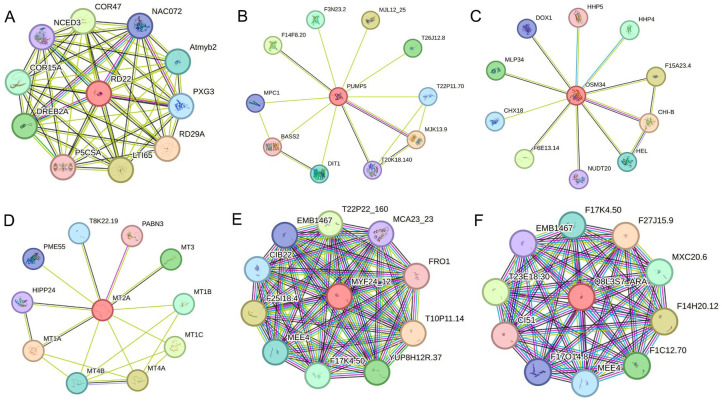
Protein interaction analysis of hub gene-encoded proteins. (**A**) BURP domain-containing protein 3-like (RD22) interaction network. (**B**) Mitochondrial uncoupling protein 5 (PUMP5) interaction network. (**C**) Thaumatin-like protein (OSM34) interaction network. (**D**) Metallothionein-like protein (MT2A) interaction network. (**E**) NADH dehydrogenase ubiquinone 1 beta subcomplex subunit (MYF24_12) interaction network. (**F**) ESSS subunit of NADH:ubiquinone oxidoreductase (Complex I) protein (Q8L3S7_ARAT) interaction network. Each node represents one protein and is labeled with the corresponding gene name in the UniProt database. Rose red lines represent known interactions experimentally determined, light bule means known interactions detected from curated databases; dark green lines indicated predicted gene neighborhood; red lines represent predicted gene fusions, dark purple means predicted gene co-occurrence; black represents co-expression, light purple indicates protein homology, and light green means others.

To further investigate their potential functions, protein–protein interaction (PPI) networks were predicted for the proteins encoded by the ten hub genes using the STRING database. Interaction networks were successfully predicted for six proteins (Figure 7). BURP domain-containing proteins have been reported to participate in pollen development in *Arabidopsis* and to regulate seed mass in cotton [32]. In *Arabidopsis*, BURP domain-containing protein 3-like localizes to the cell wall and contributes to cell enlargement [33], whereas its predicted interacting proteins (e.g., RD22) are primarily involved in low-temperature and drought stress responses. Mitochondrial uncoupling proteins regulate energy metabolism by modulating the electrochemical proton gradient and reactive oxygen species production [34], with predicted interactors mainly representing mitochondrial substrate carriers. Thaumatin-like proteins, commonly implicated, are typically involved in responses to fungal pathogens and abiotic stresses [35], their interactors are also related to both biotic and abiotic responses. Additional, metallothionein-like proteins and NADH dehydrogenases were predicted to form homomeric complexes, suggesting that they function as components of multimeric protein assemblies. These proteins are implicated in plant defense, redox regulation, and energy metabolism [36,37,38].

## 3. Discussion

In model plants, floral organ development has been extensively characterized, culminating in the ABCDE model that elucidates the genetic regulation of floral organ identity [6]. However, studies on floral development in sesame remain limited. The dehiscent-corolla mutant css1 was obtained through EMS mutagenesis. We have previously identified a candidate gene encoding GIF1 (growth-regulating factor (GRF)-Interacting factor) via a genome-wide association analysis, and its mutation is likely responsible for the dehiscent-corolla phenotype [24]. In *Arabidopsis thaliana*, GIF1 interacts with GRF proteins to regulate leaf and petal size and contributes to plant regeneration [39,40,41,42]. In our previous study, *GIF1* was significantly highly expressed in tissues of the ovary and developmental seeds of the mid–later stage, suggesting that *SiGIF1* is mainly involved in seed development in sesame. In this study, the sesame *GIF1* homolog exhibited extremely low expression (FPKM < 0.1) in both the main and lateral flowers of both genotypes, indicating that *GIF1* functions early in flower initiation, potentially within a limited population of meristematic cells. As the RNA-seq samples were collected after floral initiation, the DEGs identified here likely represent downstream targets of *GIF1*.

In sesame, the differentiation of the floral meristem and initiation of floral organ primordia occur at very early developmental stages, often before any visible morphological differences can be observed. Due to the extremely small size of early meristematic tissues and their tight enclosure within bracts, precise microdissection of floral meristems and sufficient RNA for transcriptomic analysis and reliable qRT-PCR across multiple genes and biological replicates remain technically challenging in sesame. In addition, it is also difficult to determine the specific and precise developmental period when sampling at a very early stage. Due to these biological and technical constraints, therefore, the present transcriptomic analysis was conducted at the phenotypically distinguishable stage (flowers one day before anthesis), enabling a reliable investigation of the transcriptional consequences associated with the mutant floral structure.

Interestingly, several key DEGs and hub genes identified in this study, including MADS-box genes, AP2-like transcription factors, auxin-responsive genes, and RanBP1 domain protein-encoding gene, among others, are known to function not only in early floral organ patterning but in later processes, such as petal expansion, adhesion, tissue fusion, and corolla opening. This suggests that the observed expression differences in fully developed flowers are not merely downstream effects but may represent sustained or stage-spanning regulatory roles involved in both organ differentiation and morphogenesis. Similar observations have been reported in *Rosa*, *Chrysanthemum*, and *Phalaenopsis* [43,44,45,46], where genes regulating petal adhesion and boundary formation remain active throughout late floral development, contributing to final flower morphology and dehiscence. Nevertheless, we acknowledge that examining earlier floral meristem stages would be of great value for identifying upstream regulatory events that initiate the divergence in floral organ development. Future studies will integrate laser capture microdissection (LCM), spatial transcriptomics, and stage-specific qRT-PCR to dissect gene activity during floral meristem initiation, organ primordia differentiation, and tissue adhesion establishment.

In this study, among the DEGs shared between the main and lateral flowers of Yuzhi11 and css1, two genes encoded a cyclic nucleotide-gated ion channel (CNGC) and a RanBP1 domain protein, respectively. Cyclic nucleotide-gated ion channel 4 (CNGC4) functions as a calcium channel involved in flowering time regulation in *Arabidopsis* [47], while RanBP1 participates in pistil development in tobacco through protein–protein interactions with cyclin-dependent kinases [48]. In *Arabidopsis*, mutations in *CNGC4* lead to the upregulation of miR156 and downregulation of its target gene *SQUAMOSA PROMOTER BINDING-LIKE* (*SPL*), thereby delaying blooming [47]. Although *CNGC* expression was undetectable in both the main and lateral flowers of the css1 mutant, flowering time was only slightly affected. The potential role of *CNGC*-mediated ion transport in sesame flower development thus warrants further investigation. In tobacco, RanBP1 functions in nuclear import/export during interphase and contributes to spindle checkpoint formation by interacting with *N. tabacum* cyclin-dependent kinase G;2 (NtCDKG;2), which in turn interacts with the cell cycle regulator stigma/style cell cycle inhibitor 1 (SCI1) [48]. Silencing *SCI1* increases in the size of stigmas and styles [49]. Similarly, *RanBP1* expression was absent in the main and lateral flowers of the css1 mutant, suggesting that disrupted cell division may be associated with complete corolla development. Auxin is a key regulator of floral initiation and organ differentiation [50], several key genes involved in auxin signaling and metabolic pathway are differentially expressed between css1 and Yuzhi11 (Tables S3 and S4), indicating that auxin signaling may regulate cell proliferation and expansion by influencing transport metabolism or polarity distribution, contributing to the dehiscent-corolla phenotype. Beyond that, genes involved in cytokinin, gibberellin, and ethylene signal transduction, respectively, also exhibited remarkably differential expression levels between two materials, implying that there is a complex interplay and crosstalk among plant hormones, and these diverse regulatory mechanisms enable the plant body to integrate developmental signals and environmental factors, thereby flexibly regulating morphological differences of the petals [45]. In the wild type Yuzhi11, gene expression patterns between the main and lateral flowers were highly similar, with only 312 DEGs detected, suggesting comparable developmental processes. By contrast, the css1 mutant exhibited 1921 DEGs between the main and lateral flowers, implying distinct regulatory mechanisms. This divergence likely underlies the ability of the main flowers to produce a limited number of capsules and seeds, whereas the lateral flowers fail to develop functional capsules. Several MADS-box genes associated with flower development were identified, such as *GLOBOSA* [10], *AGL62* [28], *MADS6*, and *MADS23* [29,30], along with flowering signal integrators *SOC1* and *SVP* [31]. These genes are likely involved in carpel and seed formation in the main flowers of the css1 mutant. A WGCNA further revealed two co-expression modules strongly associated with the main and lateral flowers of the mutant, from which the top five hub genes of each were identified. The co-expressed genes within these modules were enriched in metabolic pathways, highlighting their potential roles in regulating substance metabolism during floral development. Among the proteins encoded by the ten hub genes, six formed a putative interaction network in the STRING database, primarily associated with responses to biotic and abiotic stresses and energy metabolism. The hub gene *SindChr1T00222* encoded the BURP domain-containing protein 3-like protein, and some studies showed that the *BURPs* genes were a multifunctional plant-specific gene family, mainly involved in response to abiotic stress by integrating ABA signaling. In addition, *BURPs* were also included in developmental regulatory expression, though influencing the integrity of the cell wall and the patterns of cell division and differentiation, as well as regulating seed germination [51,52]. Mitochondrial uncoupling proteins, metallothionein-like proteins, NADH dehydrogenases, and NADH:ubiquinone oxidoreductase (Complex I) protein were key mediators involved in the plants mitochondrial oxidative phosphorylation system, and played important roles in ATP production, provision, and metabolic pathways during *Arabidopsis* growth and development [53,54]. In this study, these genes mentioned above were identified as key hub genes in the co-expression regulatory network regarding floral development, indicating they may function through multiple processes, such as cell division, differentiation, expansion, and growth metabolism, among others. Future functional studies, such as gene editing or overexpression analyses, are needed to elucidate the precise roles of these genes in the regulatory network governing sesame flower development.

## 4. Materials and Methods

### 4.1. Plant Materials

All plant materials used in this study were self-pollinated for more than 10 generations and were kindly preserved and supplied by Genetic Resources Center of Henan Sesame Research Center, Henan Academy of Agricultural Sciences (Zhengzhou, China). The sesame cultivar Yuzhi11 and its EMS induced dehiscent-corolla mutant, css1, were cultivated annually under standard agronomic practices at the experimental farm of Henan Sesame Research Center in Yuanyang, China (113° 97′ E and 35° 05′ N). Mutation treatment was previously conducted with a concentration of 1.0 mM EMS for 12 h according to the protocol guidelines [55]. Corolla phenotypes were observed and photographed during the full-blossom stage, approximately 50 days after sowing. Samples of root (washed free of soil), stem, leaf, main flower, lateral flower, and fruit were respectively collected, immediately frozen in liquid nitrogen, and stored at −80 °C for RNA sequencing and qRT-PCR analysis. Each sample included three biological replicates.

### 4.2. RNA Isolation and Transcriptome Sequencing

Total RNA was isolated from the collected samples using a Polysaccharide Polyphenol Total RNA Kit (Tiangen, Beijing, China) according to the manufacturer’s instructions. RNA purity, concentration, and integrity were assessed using a NanoDrop 2000/8000 microspectrophotometer (Thermo Fisher Scientific, Waltham, MA, USA), an Agilent 4200 TapeStation (Agilent, Santa Clara, CA, USA), and an Agilent RNA Screen Tape Assay (Agilent, Santa Clara, CA, USA), respectively. Samples with an RNA integrity number (RIN) ≥ 5.5 were used for RNA-seq libraries preparation using the NEBNext^®^ Ultra™ RNA Library Prep Kit for Illumina (NEB, Ipswich, MA, USA) and sequenced with 150 bp paired-end reads (PE150) on the Illumina NovaSeq X Plus platform (Annoroad Gene Technology (Beijing) Co., Ltd., Beijing, China).

### 4.3. RNA-Seq Analysis

Raw sequencing reads from 24 libraries were quality-filtered using Trimmomatic v0.39 to remove adaptors and low-quality reads. Clean reads were aligned to the *Sesamum indicum* reference genomes (accession: PRJNA315784, detailed information archived in NCBI Datasets: https://www.ncbi.nlm.nih.gov/bioproject/?term=PRJNA315784, accessed on 22 November 2024) using HiSat2 v2.1.0 [56], allowing up to two-base mismatches. The transcript abundance was quantified as fragments per kilobase of transcript per million mapped reads (FPKM) using StringTie v2.1.1. Differentially expressed genes (DEGs) between sample pairs were identified with DESeq2 v1.20.0, using a |log_2_. (fold change)| ≥ 1 and an adjusted *p*-value < 0.05 as the significant thresholds. Gene ontology (GO) and Kyoto Encyclopedia of Genes and Genomes (KEGG) pathway enrichment analyses were performed using ClusterProfiler v3.8.1, with all annotated genes in the reference genome serving as the background set.

### 4.4. Quantitative Real-Time PCR (qPCR)

A qPCR was conducted to validate the reproducibility and reliability of the RNA-Seq results. Total RNA (1 µg) from each sample was reverse transcribed using the TransScript^®^ All-in-one First-strand cDNA Synthesis SuperMix (TransGen Biotech Co., LTD., Beijing, China) following the manufacturer’s instructions. The qPCR reactions were performed with the TransStart^®^ TOP Green qPCR SuperMix (TransGen Biotech Co., LTD., China) on Roche LightCycler^®^ 480 Real-time PCR Platform (Roche Diagnostics, Basel, Switzerland). Gene expression levels of DEGs and hub genes were normalized to *SiTub1* as the internal reference. A total of 17 genes were selected for qPCR validation, and gene-specific primers were designed using the qPrimerDB (https://qprimerdb.biodb.org/analysis/, accessed on 25 May 2025). Primer sequences are listed in Table S12. Each reaction included three biological replicates and three technical replicates. Relative expression levels were calculated using the 2^−ΔΔCt^ method, the data were presented as the mean ± SD, and statistical significance was determined using the *t*-test [57]. Data were visualized using GraphPad Prism 10.

### 4.5. Weighted Gene Co-Expression Network Analysis

WGCNA R package v4.2.2 was employed to construct co-expression networks associated with the main and lateral flowers of the css1 mutant for co-expression module analysis. Genes with FPKM ≥ 1 in at least one sample and a coefficient of variation (CV) > 0.5 were retained for analysis. A hierarchical clustering dendrogram was constructed using the cutreeDynamic algorithm, with the minimum module size set to 30 genes. Modules were merged using a dynamic tree cut algorithm with a merging threshold of 0.3. Module–trait relationships were calculated and visualized in a heatmap. Based on module membership ≥ 0.9 and gene significance ≥ 0.6, the top 5 genes in each module were identified as hub genes. Co-expression networks of these hub genes were visualized using Cytoscape v3.6.1.

### 4.6. Protein–Protein Interaction Prediction

A protein–protein interaction analysis was performed using the STRING database (https://cn.string-db.org/, accessed on 17 June 2025). Interacting proteins were predicted via the “protein by sequences” tool, specifying *Arabidopsis thaliana* as the reference organism, with all other parameters set to default. The resulting protein–protein interaction networks of the hub gene-encoded proteins were visualized using Cytoscape v3.6.1.

## 5. Conclusions

This study performed a comparative transcriptomic analysis of sesame flowers between the wild-type Yuzhi11 and the dehiscent-corolla mutant css1. DEGs were identified in both the main and lateral flowers, revealing candidate genes potentially involved in floral organ development. Through a WGCNA, two gene modules were found to be significantly associated with the mutant’s main and lateral flowers, respectively. Each module contained five hub genes whose co-expression networks were further characterized. These findings provide valuable insights into the molecular mechanisms governing sesame flower and fruit development, and provide a theoretical basis for further functional studies and molecular breeding aimed at improving floral and yield-related traits.

## Figures and Tables

**Table 1:** Hub genes of the MEgreenyellow module and the MEmagenta module.

Module	Hub Genes	Annotation
MEgreenyellow	SindChr1T00222	BURP domain-containing protein 3-like
MEgreenyellow	SindChr3T02069	Pathogenesis-related protein STH-2-like
MEgreenyellow	SindChr13T01793	Mitochondrial uncoupling protein 5
MEgreenyellow	SindChr10T01595	Major allergen Pru ar 1-like
MEgreenyellow	SindChr6T01985	thaumatin-like protein
MEmagenta	SindChr5T01785	metallothionein-like protein
MEmagenta	SindChr5T01921	-
MEmagenta	SindChr3T01582	NADH dehydrogenase ubiquinone 1 beta subcomplex subunit
MEmagenta	SindChr11T01036	-
MEmagenta	SindChr4T00106	ESSS subunit of NADH: ubiquinone oxidoreductase (Complex I) protein

## Data Availability

The original contributions presented in this study are in the article/Supplementary Materials. The basic analytical data for sesame had been archived via accession number: PRJNA315784 in NCBI database, and further inquiries can be directed to the corresponding authors.

## Supplementary Materials

The following supporting information can be downloaded at: https://www.mdpi.com/article/10.3390/ijms262411841/s1.
