# Supplementary figures and images for: Comparative Transcriptomics Analysis Reveals Genes Associated with a Dehiscent-Corolla Mutant in Sesame (Sesamum indicum L.)
Source: Int J Mol Sci. 2025 Dec 8;26(24):11841. doi: 10.3390/ijms262411841 (PMC12732340; doi:10.3390/ijms262411841)

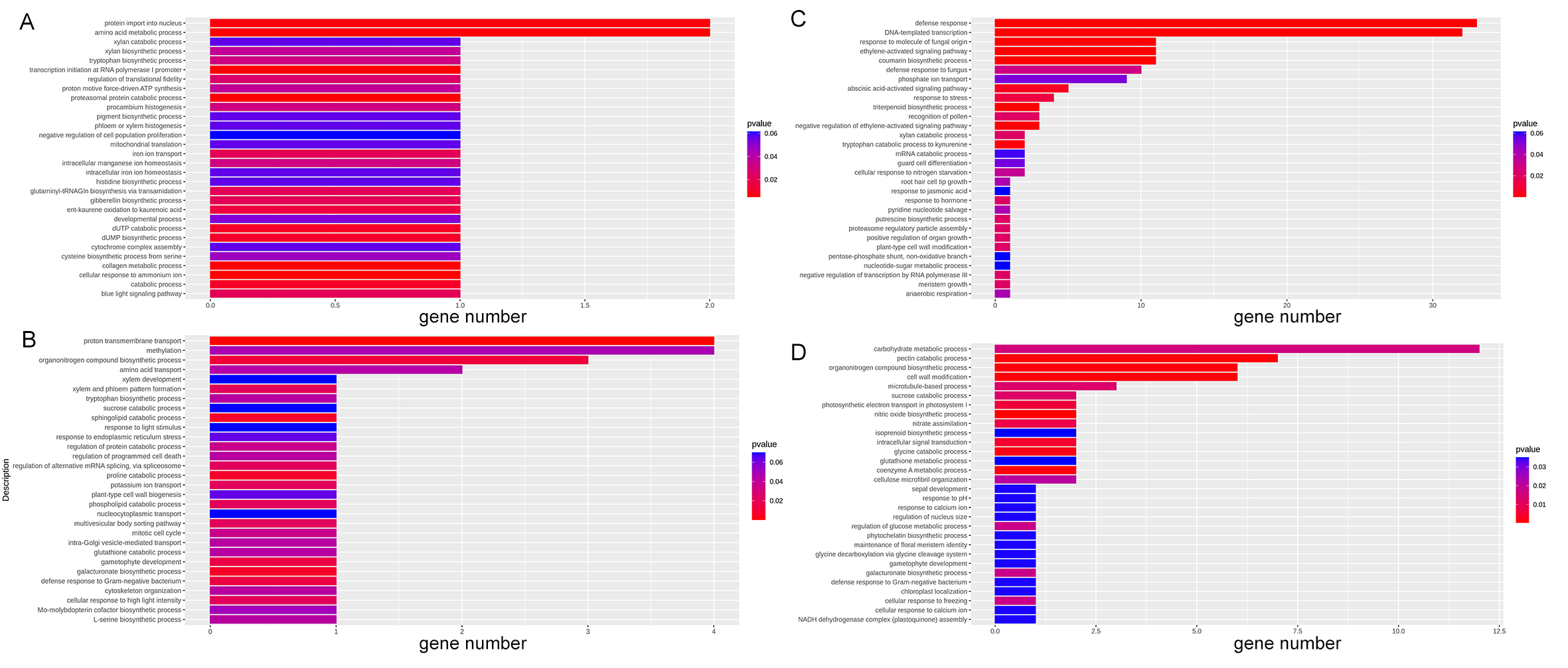

Supplement: Supplementary file 1 [file ijms-26-11841-s001.zip › Figure s1.tif]

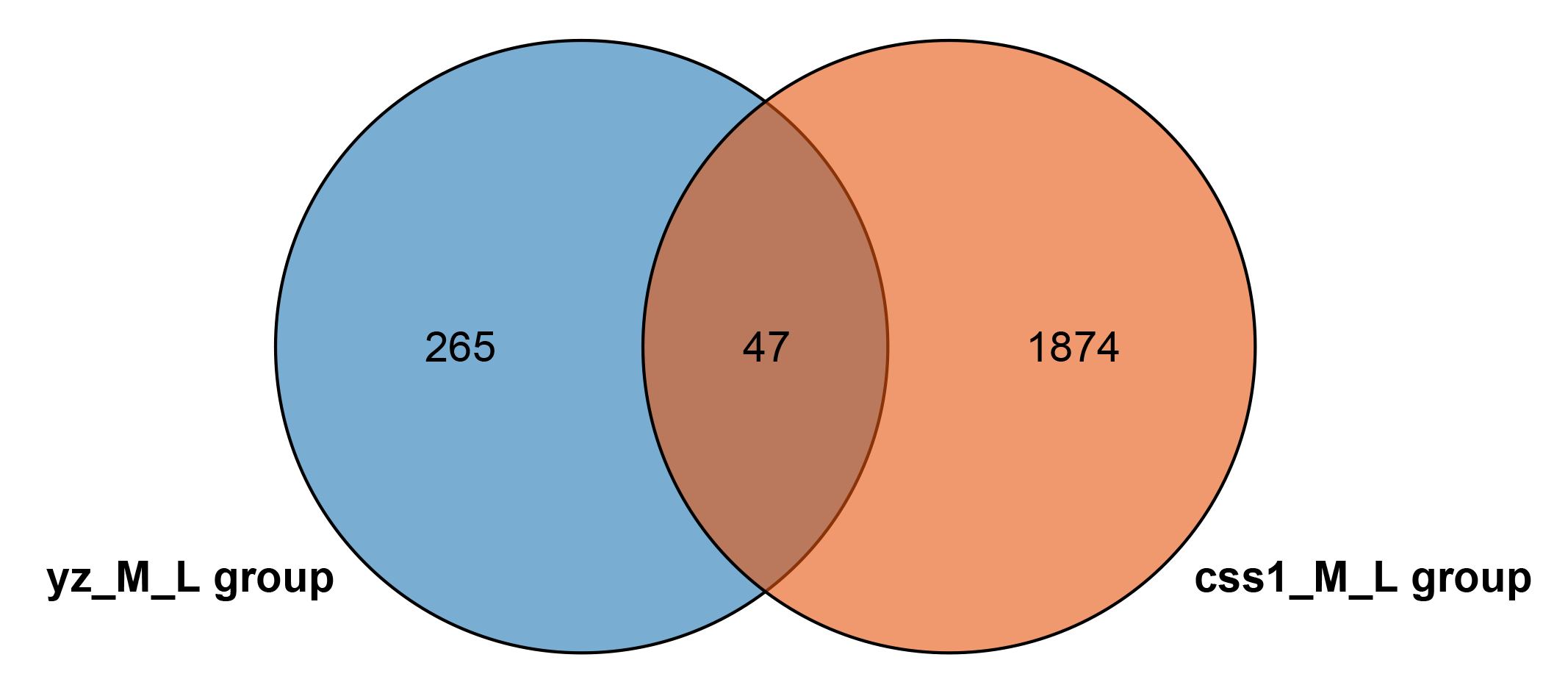

Supplement: Supplementary file 1 [file ijms-26-11841-s001.zip › Figure s2.tif]
